# Comprehensive Characterization of Flavor Compounds in Dried Goji Berry (*Lycium barbarum* L.) Obtained from Different Origins with Different Drying Methods

**DOI:** 10.3390/metabo16030183

**Published:** 2026-03-10

**Authors:** Guoli Dai, Xinru He, Bo Zhang, Linyuan Duan, Yujing Wang, Yuzhou Zhang, Huiling Ma

**Affiliations:** 1 College of Life Science, Northwest A&F University, Yangling 712100, China; 2 Institute of Goji Berry Science, Ningxia Academy of Agriculture and Forestry Sciences, Yinchuan 750002, China

**Keywords:** goji berry, drying methods, volatile compound, ethanol, 2-phenoxy-, aldehydes, ester, metabolic pathway

## Abstract

**Background:** *Lycium barbarum* L. is gaining significant interest as a medicinal and culinary raw material. The quality and aroma are significantly influenced by metabolite accumulation, which differs based on origins and drying methods. **Methods:** This study utilizes gas chromatography–mass spectrometry (GC-MS) to analyze the metabolic profiles of the ‘Ningqi’ No. 1 variety from three distinct origins employing two drying techniques (natural sun drying, NSD; hot-air drying, HAD). The samples include Zhongping, Ningxia, with HAD (1-1); Zhongning, Ningxia, with NSD (1-2); Wuwei, Gansu, with NSD (1-3); Nuomuhong, Qinghai, with NSD (1-4); and Nuomuhong, Qinghai, with HAD (1-5). **Results:** The study found that aldehydes, esters, ketones and alcohol are key secondary metabolites generated during NSD and HAD treatments of goji berry from various regions. Flavor analysis revealed the compound Ethanol, 2-phenoxy- (balsamic) was up accumulated in goji berry from Qinghai drying with NSD compared with HAD; goji berry drying with HAD collected from Ningxia compared with Qinghai; goji berry drying with NSD collected from Gansu compared with Ningxia; and goji berry drying with NSD collected from Qinghai compared with Ningxia. The compound 2-Thiophenemethanol (burnt) was up accumulated in goji berry drying with HAD collected from Ningxia compared with Qinghai. Further flavor analysis revealed that the compound Undecanal (floral) was up accumulated in goji berry drying with NSD collected from Qinghai compared with Ningxia and Gansu. 1H-Pyrrole-2-carboxaldehyde (burnt), 1-ethyl- (burnt) was up accumulated in goji berry drying with NSD collected from Qinghai compared with Gansu. KEGG enrichment analysis suggests that ‘Arginine and proline metabolism’ could be the primary metabolic pathway in the goji berry drying process. **Conclusions:** This study examined how origins and drying methods affected the metabolites and metabolic pathways of goji berries to elucidate the mechanisms impacting their quality and flavor. The findings provide important insights into the use of goji berries in functional foods and pharmaceuticals.

## 1. Introduction

*Lycium barbarum* L., a deciduous shrub of the Solanaceae family, originates from northwest of China and is globally recognized for its fruits, goji berries [1,2]. For over 2000 years, goji berries have been integral to traditional Chinese medicine and cuisine, commonly used in dishes such as soups and rice porridge [3]. It has been shown that the goji berry is rich in polysaccharides [4], polyphenols [5], carotenoids [6], betaines, amino acids, and many functional compounds [7,8]. Goji berries can boost the immune system, have antioxidant properties, affect the reproductive system, protect photoreceptor cells, and aid in cardiovascular treatment [9,10]. Clinical trials showed that daily goji berry supplements boosted macular pigment and slowed AMD (age-related macular degeneration) progression and retinal degeneration in healthy middle-aged people [11]. The cultivation of goji berries is widespread in northwestern China, including provinces like Ningxia, Gansu, Qinghai, Inner Mongolia, and Xinjiang. Zhongning County in Ningxia province is considered the traditional birthplace of goji berry farming in China.

It is widely recognized that variations in goji species result in differences in their bioactive ingredient content. *Lycium barbarum* L. and *Lycium chinense* Miller are closely related species, with *Lycium barbarum* L. comprising nearly 90% of commercially available goji berries and being the sole species included in the *Chinese Pharmacopoeia* [12]. In China, the area planted with *L. barbarum* now surpasses 134,000 hectares, with over 80% dedicated to the ‘Ningqi’ No. 1 variety, which is extensively grown in China due to its excellent quality, high productivity, and strong adaptability [12]. Prior research identified hexanal, 1-hexanol and 1-octen-3-ol as key volatile compounds in fresh red goji berries [13,14]. Yan Zheng et al. [15] identified 22 key aroma components in fresh red goji berries, such as (*E*)-β-damascenone, 1-octen-3-one, and trans-4,5-epoxy-(*E*)-2-decenal, which produce cooked apple-like, mushroom-like, and metallic odors. They also found that some key aroma components such as decenal, (*E*)-2-undecenal, (*Z*)-3-hexen-1-ol, (*E*)-2-heptenal, (*E*,*E*)-2,4- nonadienal, and methional were reduced to below detectable levels after drying. The fatty acids-derived aldehydes and ketones between raw materials and the respective heated materials also decreased. Remarkably, at the end stage of drying, the contents of some aroma components were slightly elevated, including nonanal, decanal, (*E*)-2-heptenal, (*E*)-2-undecenal, (*E,E*)-2,4-heptadienal, (*E,E*)-2,4-decedienal, and (*Z*)-3-hexen-1-ol. Fresh red goji berries, which have delicate tissues and a high moisture content of about 75–85%, are prone to microbial spoilage and decay [14]. Drying is crucial for prolonging their shelf life. Natural and hot-air drying are the prevalent methods for processing red goji berries [16]. The process of natural drying involves laying freshly harvested goji berries in a thin, flat layer under sunlight [17]. Hot-air drying offers a practical alternative to natural drying by quickly reducing surface moisture, addressing issues like weather unpredictability, contamination risks, prolonged drying times, and scalability limitations. Recently, more researchers have been exploring metabolomics studies using various drying methods. Non-targeted metabolomics analysis demonstrated significant changes in metabolite composition and pathways in peanuts due to varying drying methods [18]. A systematic metabolome analysis comparing naturally dried and hot-air-dried red goji berries has not yet been conducted.

This study utilized a GC-MS metabolomics approach to examine the metabolite profiles and relative concentrations in the ‘Ningqi’ No. 1 variety, comparing the impacts of natural sun-drying and hot-air drying methods. PCA was utilized to examine how drying methods and origins affected the metabolic profiles of ‘Ningqi’ No. 1. This study aimed to investigate the DMs of goji berries from various regions using two drying methods and identify potential marker DMs for goji berry identification, providing perspectives on various drying methods and significantly contributing to the study of key bioactive compounds influencing the quality of ‘Ningqi’ No. 1.

## 2. Materials and Methods

### 2.1. Plant Materials

Twenty goji berries (Ningqi No. 1) were used in the study, collected from various regions such as Zhongning in Ningxia, Wuwei in Gansu, and Nuomuhong in Qinghai. The detailed information of each planting site is shown in Table 1. The employed samples were of the same maturity state and roughly the same cropping pattern. The samples were divided into five groups: goji berries collected from Zhongning, Ningxia, with the HAD method (1-1); goji berries collected from Zhongning, Ningxia, with the NSD method (1-2); goji berries collected from Wuwei, Gansu, with the NSD method (1-3); goji berries collected from Nuomuhong, Qinghai, with the NSD method (1-4); and goji berries collected from Nuomuhong, Qinghai, with the HAD method (1-5). Information of the collected samples is shown in Table 2.

### 2.2. Dried Goji Berries PROCESS

**Figure 1:**
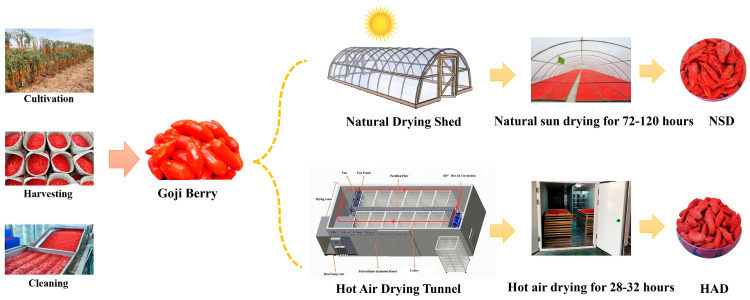
Manufacturing processes of goji berry through two different drying methods.

The detailed process was as follows (Figure 1):

(1) Harvesting: The process involved collecting unblemished, undamaged goji berries when their color changed from orange to bright red. Throughout the picking, handling, and placing processes, the fruits were treated gently to prevent impact-induced damage.
(2) Natural sun drying (NSD): Fresh goji berries are soaked in a solution with a ratio of 1:2:4:100 (ethanol, sodium ascorbate, potassium carbonate and water) for 60 s to remove wax. Then, they are evenly spread on bamboo mats with a thickness of no more than 3 cm to prevent water retention due to accumulation. The bamboo mats should be placed 30 cm above ground to create a ventilated space at the bottom and reduce the risk of local moisture and mold. During the drying process, the goji berries should not be turned over to ensure uniform heating and water loss. Choose sunny days for continuous drying for 72 to 120 h, with a moisture content of no more than 12% as the endpoint criterion for drying.
(3) Hot-air drying (HAD): After the fresh goji berries are dewaxed, they are dried in a hot-air tunnel in three stages with gradient implementation: The first stage is a high-humidity and low-temperature period, with a set temperature of 40–50 °C for 10 h, which is suitable for the high water content characteristic of goji berries in the initial drying stage. The low-temperature environment avoids the color deterioration of the fruit peel caused by high temperature and high humidity. The second stage is a medium-humidity and medium-temperature period, with the temperature raised to 50–60 °C for 12 h, which accelerates the migration rate of internal moisture to the surface of the goji berries. The third stage is a low-humidity and high-temperature period, with the temperature controlled at 60–65 °C for 8 h. At this point, a large amount of internal moisture in the goji berries has been discharged. The moderate high temperature can improve drying efficiency. The drying endpoint is determined by a moisture content of ≤12%.

All dried goji berries from the two drying methods (NSD and HAD) were ground into a powder (passed through a 425 µm sieve) and then stored at −80 °C until analysis.

### 2.3. Metabolome Analysis

#### 2.3.1. Chromatography–Mass Spectrometry Analysis

For HS-SPME (headspace-solid phase microextraction), retrieve the sample from a −80 °C freezer and pulverize it with liquid nitrogen. Thoroughly vortex and mix each sample, then weigh approximately 500 mg into a headspace vial. After pre-experimental optimization, perform headspace extraction by placing a 120 µm DVB/CWR/PDMS extraction head into the sample headspace bottle at 60 °C for 15 min, following a 5 min shaking period. Conduct analysis at 250 °C for 5 min, followed by GC-MS separation and identification. The extraction head was aged for 5 min at 250 °C in the fiber conditioning station before sampling.

#### 2.3.2. Chromatographic Conditions

A DB-5MS capillary column (30 m × 0.25 mm × 0.25 μm, Agilent J&W Scientific, Folsom, CA, USA) with high-purity helium (≥99.999%) was used as the carrier gas, maintaining a constant flow rate of 1.2 mL/min. The injection port was set to 250 °C using a no-split method and a solvent delay of 3.5 min. Initially heat the program to 40 °C for 3.5 min, then increase the temperature to 100 °C at a rate of 10 °C per minute. Increase the temperature to 180 °C at a rate of 7 °C/min; then, further raise it to 280 °C at 25 °C/min, holding this final temperature for 5 min. Mass spectra were recorded in electron impact (EI) ionization mode at 70 eV. The temperatures of the quadrupole, ion source, and interface were set to 150, 230, and 280 °C, respectively. The MS was operated in selected ion monitoring (SIM) mode for the identification and quantification of the analytes.

### 2.4. Quantitative Analysis

An autonomous database was developed using data from multiple species, the literature, partial standards, and retention indices. This database includes determined RT values and qualitative quantitative ions for precise scanning in the selection ion detection mode. Quantitative ions were chosen for integration and adjustment to improve quantification accuracy. MassHunter software (version B.05.01) was used to process the raw data from mass spectrometry for both qualitative and quantitative analysis. The samples’ metabolites were qualitatively and quantitatively analyzed using mass spectrometry, utilizing a proprietary database from Metware Metabolomics Biotechnology Co., Ltd. (Woburn, MA, USA). Adopting an internal standard semi quantitative method, a suitable standard was added to the sample during quantitative analysis, whose measured value served as a reference for calculating the content of the tested component. Based on this, select the isotopic internal standards and calculate the relative content of VOCs in the sample using the following formula:

$$X_{i}=\frac{V_{s}\times C_{s}}{M}\times \frac{I_{i}}{I_{s}}\times 10^{-3}$$

In the formula, rOAVi represents the relative odor activity value of compound i and Ci represents the relative content of the compound (μg/g or μg/mL); Ti is the threshold of the compound (Threshold, μg/g or μg/mL). Relative odor activity value (rOAV) combines compound sensory thresholds to identify key flavor compounds in food, clarifying each aroma compound’s contribution to the sample’s overall aroma characteristics. An rOAV value of 1 or greater suggests that the compound significantly influences the sample’s flavor. The calculation formula is as follows:

$$rOAV_{i}=\frac{C_{i}}{T_{i}}$$

### 2.5. Data Analysis

The statistical function prcomp in R version 4.5.2 (www.r-project.org, released on 31 October 2025) is used to perform a principal component analysis (PCA). For both groups of analyses, differential metabolites were identified by VIP (VIP > 1) and absolute Log2FC (Log2FC ≥ 1.0). KEGG metabolites’ significantly regulated pathways were analyzed using metabolite enrichment analysis (MSEA), with significance assessed via the hypergeometric test *p*-value. The VIP value, along with the score and permutation plots, is derived from the OPLS-DA results using the R package MetaboAnalystR (version 1.0.1). All samples were repeatedly measured four times. Bioinformatic analysis, including flavor radar chart, heatmap, and network diagram generation, was carried out using OmicStudio tools (available online: https://www.omicstudio.cn/toolm; accessed on 29 January 2020) [19].

## 3. Results

### 3.1. Overview of the Metabolic Profiles of Goji Berry Collected from Different Regions with Two Different Drying Methods

To assess the impact of various postharvest processes on goji berries from different regions, samples underwent two drying methods, NSD and HAD, for metabolic profiling using an untargeted metabolomics approach. The repeatability of metabolite extraction and detection can be assessed by overlapping and analyzing the total ion current (TIC) diagrams from the spectral detection of various quality control (QC) samples. The instrument’s high stability ensures data repeatability and reliability (Supplementary Figure S1).

**Figure 2:**
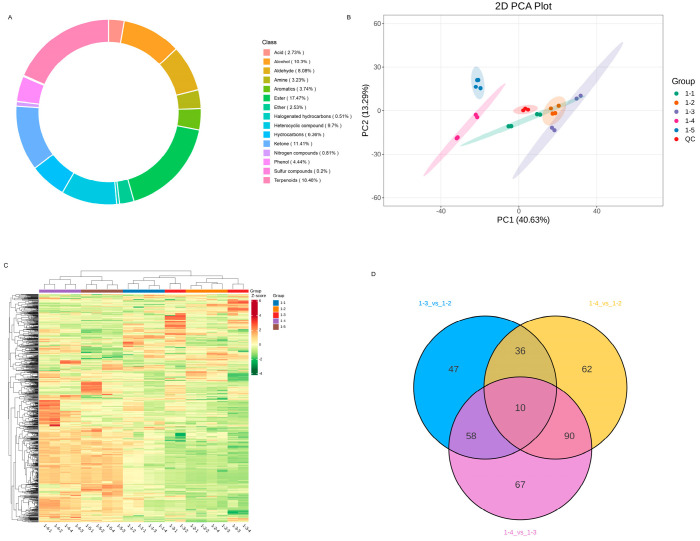
Overview of the metabolic profiles of goji berries collected from different regions with two different drying methods. (**A**) Pie diagram displaying the classification of the 990 metabolites; (**B**) the PCA score plots of the differential metabolites in all samples. (**C**) Hierarchical cluster analysis of goji berry metabolite contents among the five groups. (**D**) Venn diagram of the goji berry metabolite contents among the five groups.

In total, 990 volatile compounds were identified and divided into 15 categories including 18.48% of terpenoids, 17.47% of esters, 11.41% of ketones, 10.3% of alcohol, 9.7% of heterocyclic compounds, 8.08% of aldehydes and so on (Figure 2A). PC1 and PC2 accounted for 40.63% and 13.29% of the total variance among the samples, respectively (Figure 2B). The four replicates of the sample clustered distinctly apart from other samples. The accumulation pattern of metabolites among samples could be visualized through a heatmap hierarchical cluster analysis (Figure 2C). Furthermore, there were 10 differentially accumulated common volatile compounds in 1-3_vs_1-2, 1-4_vs_1-2 and 1-4_vs_1-3 (Figure 2D).

### 3.2. Differential Metabolites (DMs) Analysis of Goji Berry

#### 3.2.1. DMs Profiles in Two Different Drying Methods of Goji Berry

**Figure 3:**
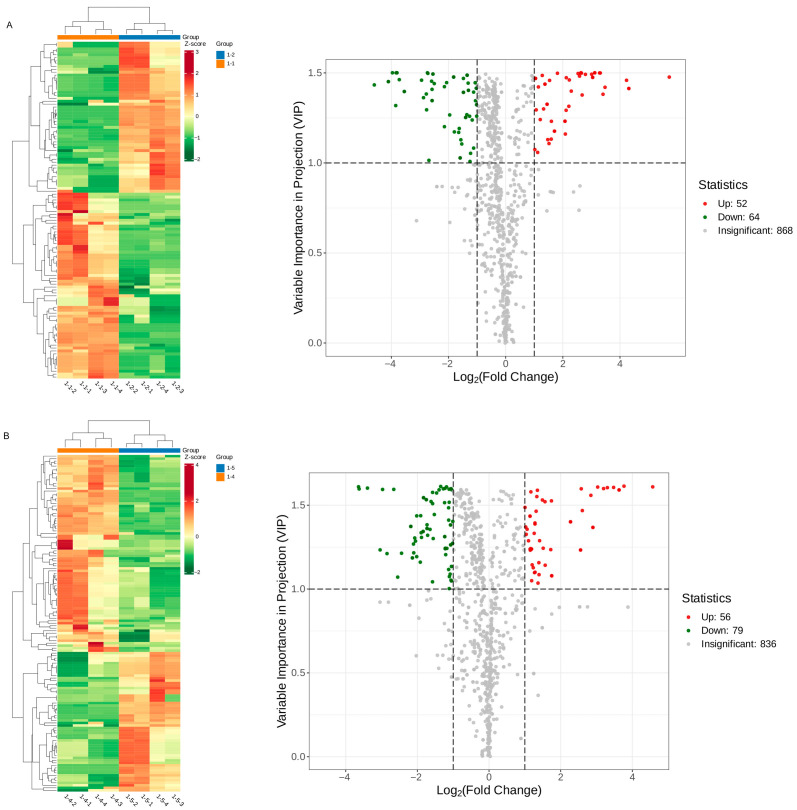
The variability of goji berry differential metabolites among two different drying methods (NSD and HAD). (**A**) The heatmap and volcano plot for 1-2 vs. 1-1; (**B**) the heatmap and volcano plot for 1-5 vs. 1-4.

**Figure 4:**
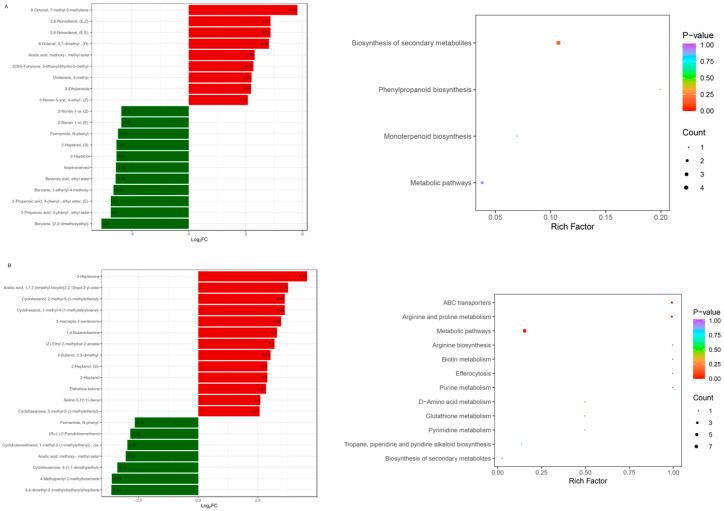
The top 20 metabolites with the highest multiples of difference, and KEGG analysis of goji berry differential metabolites among two different drying methods (NSD and HAD). (**A**) The top 20 metabolites and KEGG for 1-2 vs. 1-1; (**B**) the top 20 metabolites and KEGG for 1-5 vs. 1-4.

A heatmap analysis comparing the volatile compounds of goji berries subjected to two drying methods, NSD and HAD, revealed that certain compounds were upregulated in NSD but downregulated in HAD, indicating that drying methods influenced the volatile profile of goji berries. A total of 116 DMs were identified in 1-2 vs. 1-1. Among them, 52 volatile compounds are up accumulated and 64 are down accumulated (Figure 3A). The top 10 DMs in 1-2 vs. 1-1 are listed in Table 2 and Figure 4A. In addition, a total of 135 DMs were identified in 1-5 vs. 1-4. Among them, 56 volatile compounds are up accumulated and 79 are down accumulated (Figure 3B). The top 10 DMs in 1-5 vs. 1-4 are listed in Table 3 and Figure 4B.

In Figure 4A,B, the results showed that ‘Biosynthesis of secondary metabolite’, ‘Monoterpenoid biosynthesis’ and ‘Phenylpropanoid biosynthesis’ pathways were significantly enriched in 1-2 vs. 1-1 comparison. Meanwhile, ‘Metabolic pathways’ ‘Arginine and proline metabolism’ and ‘ABC transporters’ pathways were highly enriched in the 1-5 vs. 1-4 comparison.

#### 3.2.2. DM Profiles in Goji Berry Collected from Different Regions

**Figure 5:**
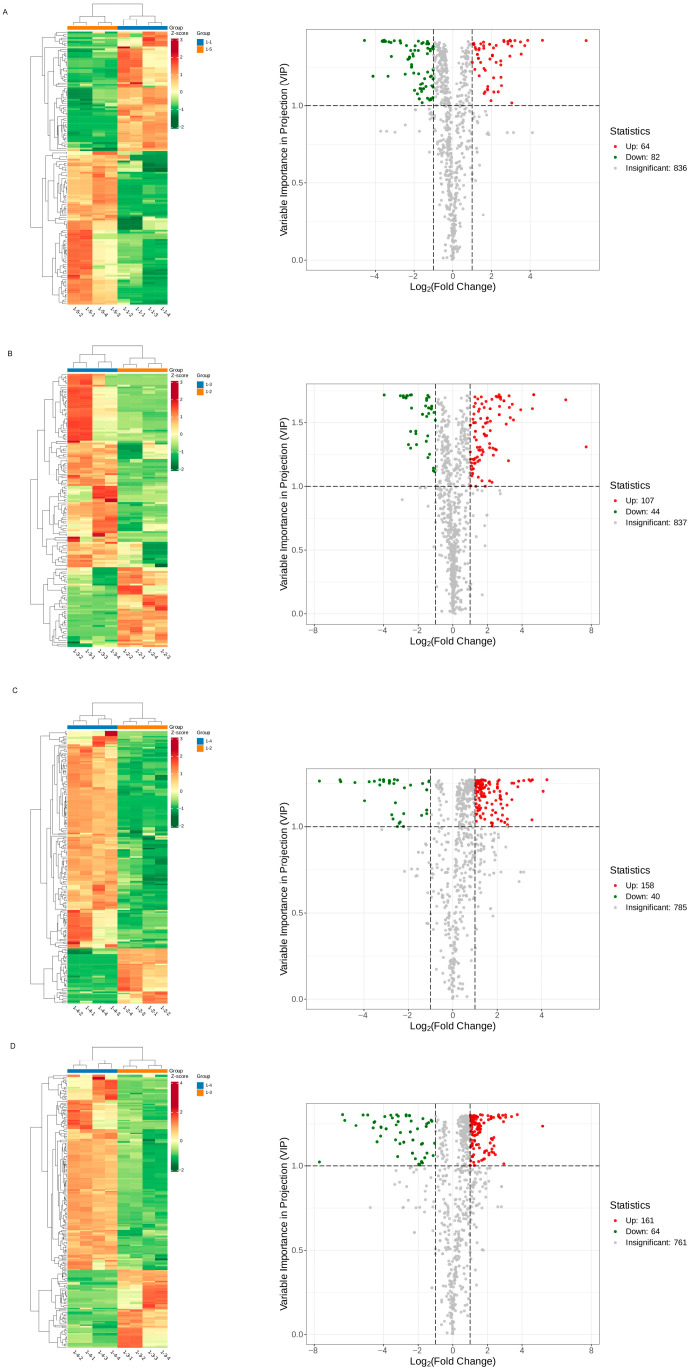
The variability of goji berry differential metabolites among three different origins. (**A**) The heatmap and volcano plot for 1-5 vs. 1-1; (**B**) the heatmap and volcano plot for 1-3 vs. 1-2; (**C**) the heatmap and volcano plot for 1-4 vs. 1-2; (**D**) the heatmap and volcano plot for 1-4 vs. 1-3.

To compare the different metabolites of goji berries between Qinghai and Ningxia under hot-air drying conditions, heatmap analysis was conducted, specifically examining samples 1-5 vs. 1-1 (Figure 5A). A total of 146 differential volatile compounds were identified between 1-1 and 1-5. The concentration of 64 volatile compounds was significantly higher in Ningxia compared with Qinghai. Similarly, Ningxia exhibited a significant reduction in 82 volatile compounds compared with Qinghai (Figure 5A). To compare the differences in the metabolites of goji berries from Qinghai, Gansu, and Ningxia under natural sun-drying conditions, a total of 151 DMs were identified in 1-3 vs. 1-2. Among them, 107 metabolites were up accumulated and 44 were down accumulated (Figure 5B). A total of 198 DMs were identified in 1-4 vs. 1-2. Among them, 158 metabolites were up accumulated and 40 were down accumulated (Figure 5C). A total of 225 DMs were identified in 1-4 vs. 1-3. Among them, 161 metabolites were up accumulated and 64 were down accumulated (Figure 5D).

**Figure 6:**
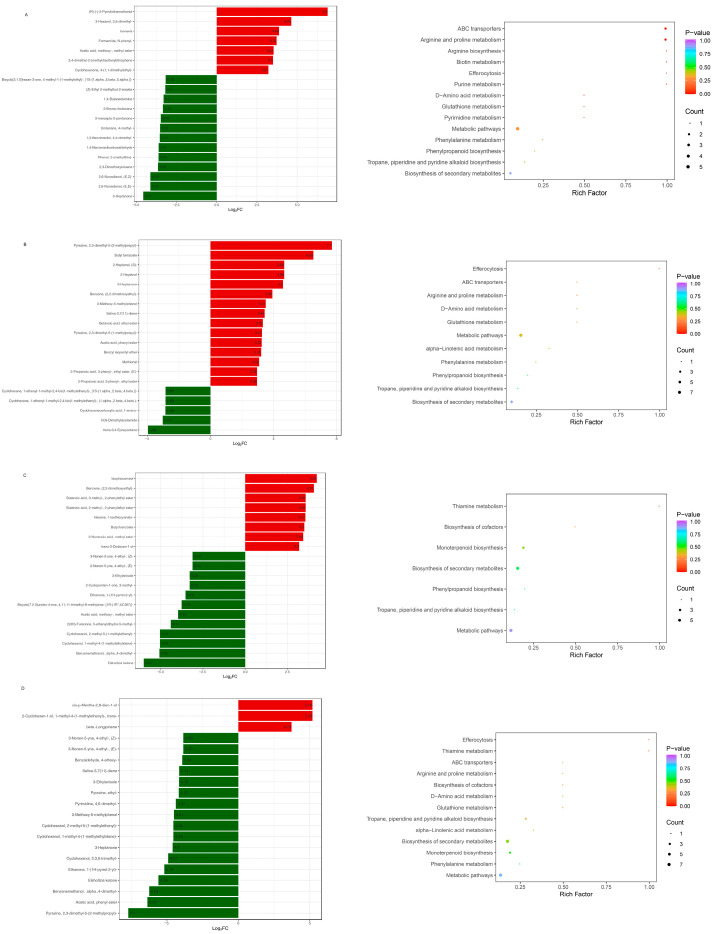
The top 20 metabolites with the highest multiples of difference, and KEGG analysis of goji berry differential metabolites among three different origins. (**A**) The top 20 metabolites and KEGG for 1-5 vs. 1-1; (**B**) the top 20 metabolites and KEGG for 1-3 vs. 1-2; (**C**) the top 20 metabolites and KEGG for 1-4 vs. 1-2; (**D**) the top 20 metabolites and KEGG for 1-4 vs. 1-3.

Metabolic pathways, ABC transporters, Arginine and proline metabolism and Biosynthesis of secondary metabolites were dominant in the 1-1 vs. 1-5 and 1-3 vs. 1-2 comparisons. Monoterpenoid biosynthesis, Thiamine metabolism and Biosynthesis of cofactors were enriched in the 1-4 vs. 1-2 comparison. Tropane, piperidine and pyridine alkaloid biosynthesis, Monoterpenoid biosynthesis and Biosynthesis of secondary metabolites were enriched in the 1-4 vs. 1-3 comparison (Figure 6).

### 3.3. Changes in Key Volatile Metabolites of Goji Berry Collected from Different Regions with Two Different Drying Methods

**Figure 7:**
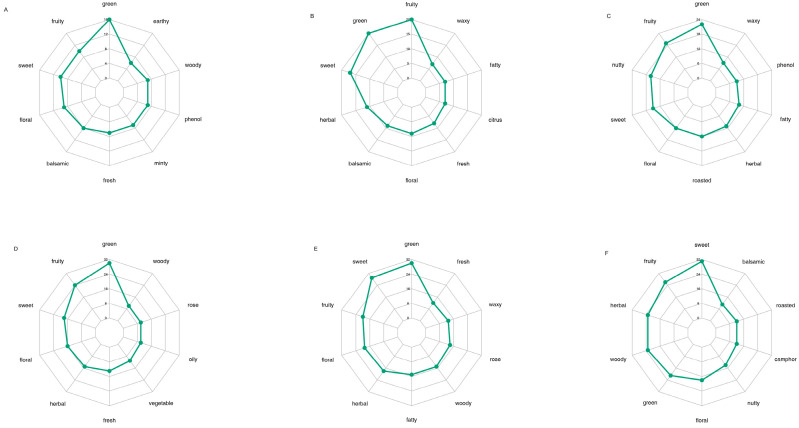
Radar chart for analysis of sensory flavor characteristics of differential metabolites. (**A**) 1-2 vs. 1-1; (**B**) 1-5 vs. 1-4; (**C**) 1-5 vs. 1-1; (**D**) 1-3 vs. 1-2; (**E**) 1-4 vs. 1-2; (**F**) 1-4 vs. 1-3.

A total of 21 volatile components that contribute to the differences in aroma among the goji berry collected from different regions with two different drying methods were identified by rOAV analysis. These components include four alcohol, three esters, three aldehydes, three amine, two heterocyclic compounds, two acids, two hydrocarbons, one ketone and one terpenoid (Table 4). When rOAV ≥ 1, the volatile components contribute to the aroma, and when rOAV > 10, they have a significant impact on the overall aroma perception [20]. A radar chart was created to display the top 10 sensory flavors with the most annotations, based on the differential volatile compounds identified according to the screening criteria for each comparison group and their annotated sensory characteristics (Figure 7). To screen the sources of aroma differences among goji berries, VIP scores were calculated for the volatile components with rOAV ≥ 10. According to the VIP score results, the contents of caprolactam (spicy) was up accumulated in the 1-4 vs. 1-2 and 1-4 vs. 1-3 comparisons; ethanone, 1-(1H-pyrrol-2-yl)- (cherry) was up accumulated in 1-1 vs. 1-5, 1-5 vs. 1-4, and 1-3 vs. 1-2; undecanal (floral) was up accumulated in the 1-4 vs. 1-2 and 1-4 vs. 1-3 comparisons; ethanol, 2-phenoxy- (balsamic) was down accumulated in 1-2 vs. 1-1 and 1-5 vs. 1-4 and up accumulated in 1-1 vs. 1-5, 1-3 vs. 1-2, and 1-4 vs. 1-2; and 1H-Pyrrole-2-carboxaldehyde (coffee) was up accumulated in 1-4 vs. 1-2.

## 4. Discussion

Volatile compound presence and concentration in goji berries are essential for their aroma, flavor, and quality. The distinct volatile organic compound (VOC) profiles of goji berries from different locations can serve as indicators of quality and support geographical indication and fair trade [21]. Analyzing volatile compound variations in goji berries from different regions offers a valuable reference for identifying their varieties and origins. Prior research employed an electronic nose model and GC-MS technique to distinguish Zhongning goji berries from other varieties [13]. Other research investigated differences in the volatile compounds of goji berries across various treatments and maturity levels [14,22]. Up to this point, numerous studies have concentrated on the health advantages of goji berries, but the drying techniques greatly affect the quality of dried goji berry products [17]. Limited research has examined the variation in volatile compounds across different regions and drying methods of goji berries. In this study, a GC-MS metabolomics approach was successfully used to detect, in all goji berry samples, a total of 990 metabolites obtained from the three origins with two different drying methods, which were identified and grouped in 15 different categories. It is apparent that goji berries have diverse and complex metabolite compositions. Additional data analysis showed that the levels and compositions of numerous metabolites varied significantly between the groups, suggesting that the drying techniques and origins of goji berry could significantly influence metabolite formation. Drying temperature is one of the key factors influencing the volatile compounds in goji berries [23]. During the drying process of goji berries, as the temperature rises, the molecular movement of volatile substances intensifies and the intermolecular interaction force weakens, making the volatile compounds more likely to evaporate from the goji berries [16]. Studies have shown that the loss rate of volatile compounds in goji berries significantly increases at higher drying temperatures. For example, in the hot-air drying experiment, when the drying temperature increased from 50 °C to 70 °C, the contents of alcohols, aldehydes, and esters, which are volatile substances in goji berries, decreased significantly [24]. This is because high temperatures accelerate the evaporation rate of these volatile substances, causing them to be lost in large quantities during the drying process. Moreover, high temperatures may also cause chemical reactions in the volatile compounds, thereby changing their types and contents [23]. Some heat-sensitive volatile compounds may undergo oxidation, decomposition, and other reactions at high temperatures, generating new substances. These new substances may have different odors and properties from the original volatile substances, thereby affecting the flavor of goji berries.

The aroma of goji berries is chiefly dependent on the proportion and levels of their volatile substances. The variety and origin of goji berries, along with their ripening stage and processing method, primarily determine the content and proportion of their volatile components [22,25,26]. Various studies have used flavor compounds to geographically distinguish fruits and crops. Peng et al. investigated volatile organic compounds (VOCs) in fresh Goji berry juice across various regions in China [27]. Zhou et al. employed headspace-GC-ion mobility spectrometry to analyze VOCs in Goji berries for distinguishing between black and red varieties [21]. A previous study identified 31 aroma components utilized to differentiate the origins of the black teas, and decanal contributed to the aroma profile of Fuyun 6 black tea [15]. Sirilertpanich et al. [28] indicated that volatolomics could successfully differentiate between the geographical origins of the same rice variety grown in regions within the same country. Qiao et al. [29] revealed that 1-Penten-3-ol, ethyl hexanoate, methyl laurate, and 2-formyltoluene were the markers of Aksu in the Xinjiang Uygur Autonomous Region, with a green and fruity aroma, and in the Shanxi province, Yuncheng city could be labeled by acetone and 2-methoxyphenol with a woody and pungent aroma. Although current techniques offer various solutions for distinguishing geographical origins and varieties, thoroughly characterizing VOCs like flavors in Goji berries to achieve precise geographical discrimination is still needed [30]. In dried goji berries, volatile compounds are predominantly alcohols, with aldehydes being the next most prevalent group. Esterases primarily generate alcohols in goji berries, functioning as solvents or carriers for producing other aromatic compounds [31]. Increased alcohol content enhances the fruity flavor of goji berries, with Propylene Glycol and Ethanol being the most prevalent alcohols in dried goji berries. It is considered an indispensable flavor component in fermented products [32]. In our study, the alcohol of Cyclobutaneethanol, 1-methyl-2-(1-methylethenyl)-, cis- in goji berries collected from Qinghai with the HAD method was down accumulated compared with that with the NSD method. Further flavor analysis revealed that the compound thanol, 2-phenoxy- was down accumulated in 1-2 vs. 1-1 and 1-5 vs. 1-4 and up accumulated in 1-1 vs. 1-5, 1-3 vs. 1-2, and 1-4 vs. 1-2. These results were consistent with the previous study [15], in which they found that decenal, (*E*)-2-undecenal, (*Z*)-3-hexen-1-ol, (*E*)-2-heptenal, (*E*,*E*)-2,4- nonadienal, and methional in fresh goji berries were reduced to below detectable levels after drying. The fatty acids-derived aldehydes and ketones between raw materials and the respective heated materials also decreased. (R)-(-)-2-Pyrrolidinemethanol and 3-Hexanol, 3,5-dimethyl- in goji berries collected from Qinghai were up accumulated compared with that collected from Ningxia. 2-Heptanol in goji berries collected from Gansu was up accumulated compared with that collected from Ningxia. The compound 2-Thiophenemethanol was down accumulated in 1-2 vs. 1-1 and up accumulated in 1-1 vs. 1-5. These compounds probably work together to create the distinctive flavor of berries sourced from various regions and processed using two distinct drying techniques.

Aldehydes, generated through lipid oxidation and the Maillard reaction, impart a fruity and oily aroma to dried goji berries due to their low odor threshold [33,34]. GC-MS analysis showed that goji berries dried at different voltages contained 18 aldehydes, with Nonanal, Hexanal, and Decanal as the dominant volatile aldehydes. Nonanal concentrations influence the aroma of Goji berries, with high levels imparting a strong oily scent and lower levels contributing rose and citrus notes. In five different goji berry juice variants, hexanal, a common aldehyde, presented a ‘green grass’ aroma and an OAV over 1. In addition, Decanal plays a major role in giving dried goji berries their fruity flavor. The compounds (E)-oct-6-enal, 7-methyl-3-methylene-, (2E,6Z)-nona-2,6-dienal, (E,Z)- and (2E,6E)-nona-2,6-dienal in goji berries collected from Ningxia with the NSD method were significantly higher than that with the HAD method. (2E,6Z)-nona-2,6-dienal and (2E,6E)-nona-2,6-dienal in goji berries collected from Ningxia with the HAD method were significantly higher than that from Qinghai. Further flavor analysis revealed that the compound Undecanal was down accumulated in the 1-5 vs. 1-4 and up accumulated in the 1-4 vs. 1-2 and 1-4 vs. 1-3 comparisons. 1-ethylpyrrole-2-carbaldehyde was down accumulated in 1-2 vs. 1-1 and up accumulated in 1-4 vs. 1-3. These compounds likely act synergistically to establish the signature flavor of goji berries collected from different regions with different drying methods.

Amino acid metabolite variations, due to different drying methods, are typically linked to the intricate biochemical reactions of proteins in goji berries. During the drying phase, endogenous enzymes and microorganisms hydrolyze proteins into smaller peptides and amino acids [35]. As one of the largest and most ancient protein families, ABC transporters are vital for transporting crucial molecules and significantly contribute to important biological processes in plants [36]. ABC transporters play a role in moving various substrates across membranes, such as amino acids, vitamins, sugars, lipids, metal ions, and secondary metabolites [37]. Data analyses revealed that the metabolic pathways ‘Arginine and proline metabolism’ and ‘ABC transporters’ were significantly upregulated under HAD treatment compared with NSD, with an increase in metabolites associated with amino acid biosynthesis.

Ester compounds impart a pleasant fruity flavor to dried goji berries due to their low threshold. The higher the content, the stronger the fruity flavor. Earlier research used GC-MS to identify the volatile compounds in African goji berries (*Vangueria infausta* L.) and discovered that the aroma of this goji type primarily came from ethyl caproate and ethyl caprylate [38]. In the present study, the compounds Acetic acid, methoxy-, methyl ester; Butanoic acid, ethyl ester; Benzene, 1-ethenyl-4-methoxy-2-Propenoic acid; 2-Propenoic acid, 3-phenyl-, ethyl ester; and (E)- Benzene, (2,2-dimethoxyethyl)- in goji berries collected from Ningxia with the HAD method were significantly higher than that with the NSD method. The compounds Acetic acid, methoxy-, methyl ester and 4-Methylpentyl 2-methylbutanoate in goji berries collected from Qinghai with HAD were significantly higher than that with the NSD method. Acetic acid, methoxy-, methyl ester; Butanoic acid, 2-methyl-, 2-phenylethyl ester; Butanoic acid, 3-methyl-, 2-phenylethyl ester and Hexane, 1-isothiocyanato- in goji berries collected from Qinghai was significantly higher than that from Ningxia. Butyl benzoate in goji berries collected from Gansu was significantly higher than that from Ningxia. Flavor analysis showed that Butanoic acid, 3-methyl-, 3-methylbutyl ester was up accumulated in 1-5 vs. 1-4, which exhibited the sweet and fruity flavors.

However, this study has potential limitations. Firstly, the HAD treatment samples from Gansu were accidentally contaminated during the drying process, ultimately resulting in the failure of these samples to meet the testing requirements. This has affected the completeness and standardization of the related analysis. To draw a clear conclusion, further investigation is necessary. Secondly, although we investigated representative regions of China producing goji berries, the number of sampling points is relatively small. Future research should use more sampling points to validate the results of this study.

## 5. Conclusions

The study investigated the metabolic profiles of goji berry collected from different regions with two different drying methods. In the dry processing of goji berries, 15 categories of secondary metabolites were identified, with terpenoids, esters, ketones, and alcohols being particularly significant. Flavor analyses revealed that the compound Ethanol, 2-phenoxy- (balsamic) was up accumulated in goji berry from Qinghai drying with NSD compared with HAD; goji berry drying with HAD collected from Ningxia compared with Qinghai; goji berry drying with NSD collected from Gansu compared with Ningxia; and goji berry drying with NSD collected from Qinghai compared with Ningxia. The compound 2-Thiophenemethanol (burnt) was up accumulated in goji berry drying with HAD collected from Ningxia compared with Qinghai. KEGG enrichment analyses identified 16 pathways, suggesting ‘Arginine and proline metabolism’ as a potentially relevant metabolite pathway for the goji berry drying process. This study investigates how origins and drying methods affect the metabolites and metabolic pathways of goji berries, with the goal of understanding the mechanisms that influence their quality and flavor. The findings provide important insights for the use of goji berries in functional foods and pharmaceuticals.

## Figures and Tables

**Table 1:** Climatic and geographical information of sampling points of goji berry.

Test Site	Longitude	Latitude	Elevation (m)	Area (acre)	Precipitation (mm)	Temperature Equalization	Sunshine Duration
Zhongning, Ningxia	105°38′12″	37°29′54″	1291	30	170	10.7	2360
Wuwei, Gansu	102°51′3″	37°39′41″	1761	5	220	8.6	2580
Nuomuhong, Qinghai	97°22′28″	37°21′18″	2846	143	40	6.5	2500

**Table 2:** The information of collected goji berries.

Species	Tissue	Origin	Drying Method	Number of Replicates
*Lycium barbarum*	fruits	Zhongning, Ningxia	HAD method	4
			NSD method	4
*Lycium barbarum*	fruits	Wuwei, Gansu	NSD method	4
*Lycium barbarum*	fruits	Nuomuhong, Qinghai	HAD method	4
			NSD method	4

**Table 3:** Identification of top 10 differentially abundant metabolites in all comparisons.

Group	Compounds	Retention Indices	Deviation Values	VIP	Class I	Log_2_ FC	Regulation
	7-methyl-3-methylideneoct-6-enal	1160.2	13.2	1.476942	Aldehyde	5.724995	up
	(2E,6Z)-nona-2,6-dienal	1162.24	7.49	1.413455	Aldehyde	4.309339	up
	(2E,6E)-nona-2,6-dienal	1160.91	7.91	1.413455	Aldehyde	4.309339	up
	(3R)-3,7-dimethyloct-6-enal	1160.16	7.16	1.459067	Terpenoids	4.229467	up
1-2 vs. 1-1	Methyl 2-methoxyacetate	971.89	6.89	1.420258	Ester	3.476692	up
	Ethyl butanoate	794.42	−7.95	1.318723	Ester	−3.84764	down
	1-ethenyl-4-methoxybenzene	1164.67	8.67	1.500791	Ether	−3.96282	down
	Ethyl (E)-3-phenylprop-2-enoate	1476.96	5.8	1.451734	Ester	−4.10466	down
	Ethyl (E)-cinnamate	1457.41	−5.59	1.451734	Ester	−4.10466	down
	2,2-dimethoxyethylbenzene	1218.65	−3.35	1.433342	Ether	−4.59709	down
	3-Heptanone	890.69	3.69	1.60921	Ketone	4.568937	up
	(1,7,7-trimethyl-2-bicyclo[2.2.1]heptanyl) acetate	1295.64	7.64	1.61349	Ester	3.767919	up
	2-methyl-5-prop-1-en-2-ylcyclohexan-1-ol	1201.46	5.46	1.590945	Terpenoids	3.630261	up
	1-methyl-4-propan-2-ylidenecyclohexan-1-ol	1345.6	4.6	1.590945	Terpenoids	3.630261	up
1-5 vs. 1-4	3-sulfanylpentan-2-one	889.74	−12.26	1.60598	Ketone	3.477655	up
	2-(1-methyl-2-prop-1-en-2-ylcyclobutyl)ethanol	1206.6	−12.4	1.593843	Alcohol	−2.97168	down
	Methyl 2-methoxyacetate	971.89	6.89	1.233495	Ester	−3.04074	down
	4-tert-butylcyclohexan-1-one	1206.63	−1.37	1.601909	Ketone	−3.39452	down
	4-Methylpentyl 2-methylbutanoate	1206.29	9.29	1.59688	Ester	−3.62715	down
	3,4-dimethyl-2-(methyldisulfanyl)thiophene	1492.05	−7.95	1.609939	Heterocyclic compound	−3.64313	down
	[(2R)-pyrrolidin-2-yl]methanol	973.05	−8.95	1.421744	Alcohol	6.89757	up
	3,5-dimethylhexan-3-ol	873.41	−9.59	1.42354	Alcohol	4.636808	up
	Isoneral	1164.9	−3.1	1.423122	Aldehyde	3.875479	up
	N-phenylformamide	1217.67	−3.33	1.389067	Amine	3.723096	up
1-5 vs. 1-1	Methyl 2-methoxyacetate	971.89	6.89	1.334295	Ester	3.53815	up
	2-methylsulfanylphenol	1162.29	−3.91	1.421495	Phenol	−3.61741	down
	1,2-dimethoxy-3-methylbenzene	1161.99	−10.01	1.417967	Ether	−3.65197	down
	(2E,6Z)-nona-2,6-dienal	1162.24	7.49	1.191889	Aldehyde	−4.12627	down
	(2E,6E)-nona-2,6-dienal	1160.91	7.91	1.191889	Aldehyde	−4.12627	down
	Heptan-3-one	890.69	3.69	1.422688	Ketone	−4.56894	down
	2,3-dimethyl-5-(2-methylpropyl)pyrazine	1190.99	0.99	1.310962	Heterocyclic compound	7.704746	up
	Butyl benzoate	1381.1	4.1	1.680439	Ester	6.526848	up
	Heptan-2-ol	899.2	−1.16	1.72042	Alcohol	4.676962	up
	(2S)-heptan-2-ol	899.16	−3.84	1.72042	Alcohol	4.676962	up
1-3 vs. 1-2	Heptan-3-one	890.69	3.69	1.610298	Ketone	4.599624	up
	(1R,2R,4S)-1-ethenyl-1-methyl-2,4-bis(prop-1-en-2-yl)cyclohexane	1398.78	0.78	1.699443	Terpenoids	−2.84764	down
	(1S,2R,4S)-1-ethenyl-1-methyl-2,4-bis(prop-1-en-2-yl)cyclohexane	1398.78	7.78	1.699443	Terpenoids	−2.84764	down
	1-aminocyclohexane-1-carboxylic acid	1398.95	4.95	1.712249	Acid	−2.85699	down
	N,N-Dimethylacetamide	862.39	−5.87	1.712147	Amine	−3.02581	down
	(2S,3S)-2-butyl-3-ethyloxirane	924.04	−7.96	1.718457	Heterocyclic compound	−3.96275	down
	Isopinocarveol	1165.16	−12.84	1.272144	Terpenoids	4.239267	up
	2,2-dimethoxyethylbenzene	1218.65	−3.35	1.205743	Ether	4.068944	up
	2-phenylethyl 2-methylbutanoate	1491.24	3.24	1.271458	Ester	3.582891	up
	2-phenylethyl 3-methylbutanoate	1491.24	0.24	1.271458	Ester	3.582891	up
1-4 vs. 1-2	1-isothiocyanatohexane	1196.79	6.79	1.038799	Ester	3.565584	up
	5-ethenyl-5-methyloxolan-2-one	1042.06	−0.94	1.255816	Ketone	−4.40654	down
	2-methyl-5-prop-1-en-2-ylcyclohexan-1-ol	1201.46	5.46	1.26107	Terpenoids	−5.0466	down
	1-methyl-4-propan-2-ylidenecyclohexan-1-ol	1345.6	4.6	1.26107	Terpenoids	−5.0466	down
	1-(4-methylphenyl)ethanol	1118.31	0.31	1.271704	Alcohol	−5.08006	down
	Elsholtzia ketone	1201.53	−0.47	1.264379	Ketone	−6.00705	down
	(1R,4S)-1-methyl-4-prop-1-en-2-ylcyclohex-2-en-1-ol	1120.8	−12.2	1.235211	Terpenoids	5.189587	up
	(1S,4S)-1-methyl-4-prop-1-en-2-ylcyclohex-2-en-1-ol	1120.8	−2.2	1.235211	Alcohol	5.189587	up
	2,6,6-trimethyl-9-methylidenetricyclo[5.4.0.02,8]undecane	1391.42	−11.58	1.304643	Terpenoids	3.726381	up
	4-tert-butylcyclohexan-1-one	1206.63	−1.37	1.293154	Ketone	3.394523	up
1-4 vs. 1-3	2-(2-methylpropyl)pyrazine	1035.02	−7.98	1.289073	Heterocyclic compound	3.056132	up
	1-(1H-pyrrol-2-yl)ethanone	1064.6	1.6	1.302654	Heterocyclic compound	−5.18102	down
	Elsholtzia ketone	1201.53	−0.47	1.239136	Ketone	−5.59369	down
	1-(4-methylphenyl)ethanol	1118.31	0.31	1.270427	Alcohol	−6.23794	down
	Phenyl acetate	1063.15	1.17	1.304682	Ester	−6.36593	down
	2,3-dimethyl-5-(2-methylpropyl)pyrazine	1190.99	0.99	1.022955	Heterocyclic compound	−7.70475	down

**Table 4:** Volatile components with rOAV ≥ 10 of goji berry.

Compounds	Primary Classification	CAS	Threshold	Odor
1,2-Cyclohexanedione	Ketone	765-87-7	450	sweet, acorn, nut skin, maple, caramel, brothy
Cis-Nerolidol	Terpenoids	3790-78-1	64	waxy, floral
Caprolactam	Amine	105-60-2	59.7	amine, spicy
Ethanone, 1-(1H-pyrrol-2-yl)-	Heterocyclic compound	1072-83-9	170	musty, nut skin, maraschino, cherry, coumarin, licorice, walnut, bread
Cyclohexaneacetic acid	Acid	5292-21-7	19.1	sharp, acetic, fatty, cheese, musty, powdery, honey, caramel
Dodecane	Hydrocarbons	112-40-3	10	alkane
Pentadecane	Hydrocarbons	629-62-9	13,000	waxy
2-Methyl-3-furanthiol	Alcohol	28588-74-1	160	sulfury, meaty, fishy, metallic
2-Acetyl-5-methylfuran	Heterocyclic compound	1193-79-9	40.87	strong, musty, nutty, hay, coconut, coumarin, milky
Undecanal	Aldehyde	112-44-7	12.5	waxy, soapy, floral, aldehydic, citrus, green, fatty, fresh
2H-Pyran-2-one, 6-hexyltetrahydro-	Ester	710-04-3	19	creamy, fatty, coconut, fruity, peach, waxy
Ethanol, 2-phenoxy-	Alcohol	122-99-6	690	mild, rose, balsamic, cinnamyl
1H-Pyrrole-2-carboxaldehyde	Aldehyde	1003-29-8	65	musty, beefy, coffee
2-Thiophenemethanol	Alcohol	636-72-6	15	ethereal, fermented, burnt, alliaceous, coffee, savory
N,N-Dimethylacetamide	Amine	127-19-5	170	ammoniacal
1H-Pyrrole-2-carboxaldehyde, 1-ethyl-	Aldehyde	2167-14-8	65	burnt, roasted peanut
Hexanoic acid, 2-ethyl-	Acid	149-57-5	27	paint, varnish
Benzenemethanol, .alpha.,4-dimethyl-	Alcohol	536-50-5	42.7	sweet, hawthorn, floral, nutty, powdery
1,4-Butanediamine	Amine	110-60-1	22	animalic, rotten, fishy
n-Amyl isovalerate	Ester	25415-62-7	12	apple, fresh fruit
Butanoic acid, 3-methyl-, 3-methylbutyl ester	Ester	659-70-1	19.80717	sweet, fruity, green, ripe apple, jammy, tropical

## Data Availability

The original contributions presented in this study are included in the article/Supplementary Materials. Further inquiries can be directed to the corresponding author.

## Supplementary Materials

The following supporting information can be downloaded at https://www.mdpi.com/article/10.3390/metabo16030183/s1. Figure S1: The mass spectrometry analysis of the total ion and the TIC overlap of the QC sample mass spectrum.

## References

[1] Lei Z.L., Chen X.Q., Cao F.L., Guo Q.R., Wang J.H. (2022). Phytochemicals and bioactivities of Goji (*Lycium barbarum* L. and *Lycium chinense* Mill.) leaves and their potential applications in the food industry: A review. Int. J. Food Sci. Technol..

[2] Yu C.X., Chen Y.H., Ahmadi S., Wu D.M., Wu J.X., Ding T., Liu D.H., Ye X.Q., Chen S.G., Pan H.B. (2023). Goji berry leaf exerts a comparable effect against colitis and microbiota dysbiosis to its fruit in dextran-sulfate-sodium-treated mice. Food Funct..

[3] Kulczynski B., Gramza-Michalowska A. (2016). Goji Berry (*Lycium barbarum*): Composition and Health Effects—A Review. Pol. J. Food Nutr. Sci..

[4] Xie J.H., Tang W., Jin M.L., Li J.E., Xie M.Y. (2016). Recent advances in bioactive polysaccharides from *Lycium barbarum* L., *Zizyphus jujuba* Mill, *Plantago* spp., and *Morus* spp.: Structures and functionalities. Food Hydrocoll..

[5] Magiera S. (2015). Chromatographic Determination of Phenolic Acids and Flavonoids in *Lycium barbarum* L. and Evaluation of Antioxidant Activity. Food Anal. Methods.

[6] Hempel J., Schädle C.N., Sprenger J., Heller A., Carle R., Schweiggert R.M. (2017). Ultrastructural deposition forms and bioaccessibility of carotenoids and carotenoid esters from goji berries (*Lycium barbarum* L.). Food Chem..

[7] Amagase H., Farnsworth N.R. (2011). A review of botanical characteristics, phytochemistry, clinical relevance in efficacy and safety of *Lycium barbarum* fruit (Goji). Food Res. Int..

[8] Zhang Q.Y., Chen W.W., Zhao J.H., Xi W.P. (2016). Functional constituents and antioxidant activities of eight Chinese native goji genotypes. Food Chem..

[9] Zhu Y.F., Zhao Q.P., Gao H., Peng X.D., Wen Y.M., Dai G.D. (2016). *Lycium barbarum* polysaccharides attenuates N-methy-N-nitrosourea-induced photoreceptor cell apoptosis in rats through regulation of poly (ADP-ribose) polymerase and caspase expression. J. Ethnopharmacol..

[10] Tang H.L., Chen C., Wang S.K., Sun G.J. (2015). Biochemical analysis and hypoglycemic activity of a polysaccharide isolated from the fruit of *Lycium barbarum* L.. Int. J. Biol. Macromol..

[11] Chen X., Wei D.D., Lin M., Wang X.S., Kang H.J., Ni L., Qian D.W., Guo S., Duan J.A. (2024). Comparative evaluation of four *Lycium barbarum* cultivars on NaIO3-induced retinal degeneration mice via multivariate statistical analysis. J. Ethnopharmacol..

[12] Yao R., Heinrich M., Weckerle C.S. (2018). The genus Lycium as food and medicine: A botanical, ethnobotanical and historical review. J. Ethnopharmacol..

[13] Lu J.F., Li H.X., Quan J.P., An W., Zhao J.H., Xi W.P. (2017). Identification of characteristic aroma volatiles of Ningxia goji berries (*Lycium barbarum* L.) and their developmental changes. Int. J. Food Prop..

[14] Xie L., Mujumdar A.S., Zhang Q., Wang J., Liu S.X., Deng L.Z., Wang D., Xiao H.W., Liu Y.H., Gao Z.J. (2017). Pulsed vacuum drying of wolfberry: Effects of infrared radiation heating and electronic panel contact heating methods on drying kinetics, color profile, and volatile compounds. Dry. Technol..

[15] Zheng Y., Oellig C., Zhu L., Bauer V., Vetter W., Zhang Y.Y. (2025). Revealing the key aroma codes and (furan) fatty acids in fresh red goji berries and the impacts of the hot-air drying process. Food Chem..

[16] Batu H.S., Kadakal Ç. (2021). Drying characteristics and degradation kinetics in some parameters of goji berry (*Lycium barbarum* L.) fruit during hot air drying. Ital. J. Food Sci..

[17] Cui C.J., Zhao D.D., Huang J., Hao J.X. (2022). Progress on research and development of goji berry drying: A review. Int. J. Food Prop..

[18] Zhang T.T., Zhao X.Y., Bo J.X., Zhu W.X., Chen P.X., Jiang M.M., Chen G.Q. (2025). Comprehensive analysis of changes in the drying characteristics, quality, and metabolome of peanuts by different drying methods. Food Res. Int..

[19] Lyu F., Han F.R., Ge C.L., Mao W.K., Chen L., Hu H.P., Chen G.G., Lang Q.L., Fang C. (2023). OmicStudio: A composable bioinformatics cloud platform with real-time feedback that can generate high-quality graphs for publication. Imeta.

[20] Ye G.D., Guan L.N., Zhang M., Li S.X., Mi Y.J. (2025). Analysis of key differential aroma compounds in thirty Japonica rice cultivars from Northeast China by integrating GC-O-MS, OAV, and chemometrics. J. Food Compos. Anal..

[21] Zhou Y.X., Wang D.D., Duan H., Zhou S.Q., Guo J.H., Yan W.J. (2023). Detection and analysis of volatile flavor compounds in different varieties and origins of goji berries using HS-GC-IMS. LWT-Food Sci. Technol..

[22] Oguz I., Oguz H.I., Ürün I., Attar S.H., Atasever S., Kafkas N.E. (2023). Determination of Aroma and Protein Contents in Organic *Lycium barbarum* L. and Lycium chinense Miller Fruits in Different Ripening Periods. Erwerbs-Obstbau.

[23] Do S., Kim Y., Yim J., Lee K.G. (2024). Analysis of volatile compounds, betaine, and antioxidant effect in goji berry (*Lycium barbarum* L.) powder extracted by various drying methods and extraction solvents. Curr. Res. Food Sci..

[24] Ding K.K., Wu P.J., Li B.T., Jiang F.L., Sun B.X. (2026). Characterization of hot air drying behavior and dynamic moisture prediction in goji berries using LF NMR. J. Food Compos. Anal..

[25] Hao J., Dong F.J., Li Y.L., Wang S.L., Cui J.R., Zhang Z.F., Wu K.N. (2022). Investigation of the data fusion of spectral and textural data from hyperspectral imaging for the near geographical origin discrimination of wolfberries using 2D-CNN algorithms. Infrared Phys. Technol..

[26] Lopatriello A., Previtera R., Pace S., Werner M., Rubino L., Werz O., Taglialatela-Scafati O., Forino M. (2017). NMR-based identification of the major bioactive molecules from an Italian cultivar of *Lycium barbarum*. Phytochemistry.

[27] Peng Q., Huang J.X., Li S.S., Massou B.B., Chen Z.Y., Zhu Q., Xie G.F. (2024). Comprehensive origin authentication of wolfberry pulp (*Lycium barbarum* L.) using multimodal sensory analysis and chemometrics. Ind. Crops Prod..

[28] Sirilertpanich P., Ekkaphan P., Andriyas T., Leksungnoen N., Ruengphayak S., Vanavichit A., De-Eknamkul W., Tansawat R. (2024). Metabolomics study on the main volatile components of Thai colored rice cultivars from different agricultural locations. Food Chem..

[29] Qiao Y.N., Bi J.F., Chen Q.Q., Wu X.Y., Gou M., Hou H.N., Jin X.W., Purcaro G. (2021). Volatile Profile Characterization of Winter Jujube from Different Regions via HS-SPME-GC/MS and GC-IMS. J. Food Qual..

[30] Han L., Zou W.T., Wang W.X., Wang L.H., Liu P.P., Tang L.H., Lv Y., Yu Y.J., She Y.B. (2025). Comprehensive characterization of flavor compounds in goji berry by HS-SPME-GCMS combined with AntDAS-GCMS for geographical discrimination. Food Chem.-X.

[31] Meng Z.F., Cui X.N., Liu Y., Hu R.S., Du C.Y., Wang S., Zhang F. (2022). Drying characteristics of banana slices under heat pump-electrohydrodynamic (EHD) combined drying. Sustain. Energy Technol. Assess..

[32] Zhang J., Ding C.J., Lu J.L., Wang H.X., Bao Y.T., Han B.Y., Zhu J., Duan S.S., Song Z.Q., Chen H. (2024). Effects of electrohydrodynamics on drying characteristics and volatile profiles of goji berry (*Lycium barbarum* L.). LWT-Food Sci. Technol..

[33] Xu L., Zang E.H., Sun S.Y., Li M.H. (2023). Main flavor compounds and molecular regulation mechanisms in fruits and vegetables. Crit. Rev. Food Sci. Nutr..

[34] Hu Y.Y., Zhao G.H., Yin F.W., Liu Z.Y., Wang J.L., Qin L., Zhou D.Y., Shahidi F., Zhu B.W. (2022). Effects of roasting temperature and time on aldehyde formation derived from lipid oxidation in scallop (Patinopecten yessoensis) and the deterrent effect by antioxidants of bamboo leaves. Food Chem..

[35] Zhang L.C., Hao N., Li W.J., Zhang B.Q., Shi T.Y., Xie M.X., Yu M. (2022). Effect of Ultrasonic Induction on the Main Physiological and Biochemical Indicators and γ-Aminobutyric Acid Content of Maize during Germination. Foods.

[36] Rees D.C., Johnson E., Lewinson O. (2009). ABC transporters: The power to change. Nat. Rev. Mol. Cell Biol..

[37] How S.S., Nathan S., Lam S.D., Chieng S. (2025). ATP-binding cassette (ABC) transporters: Structures and roles in bacterial pathogenesis. J. Zhejiang Univ.-Sci. B.

[38] Raice R.T., Sjoholm I., Wang H.L., Bergenstahl B. (2015). Characterization of volatile components extracted from Vangueria infausta (African medlar) by using GC-MS. J. Essent. Oil Res..

