# Supplementary material for: Comprehensive Characterization of Flavor Compounds in Dried Goji Berry (Lycium barbarum L.) Obtained from Different Origins with Different Drying Methods
Source: Metabolites. 2026 Mar 10;16(3):183. doi: 10.3390/metabo16030183 (PMC13027905; doi:10.3390/metabo16030183)
Supplement: Supplementary file 1 [file metabolites-16-00183-s001.zip › metabolites-4079335-supplementary.pdf]

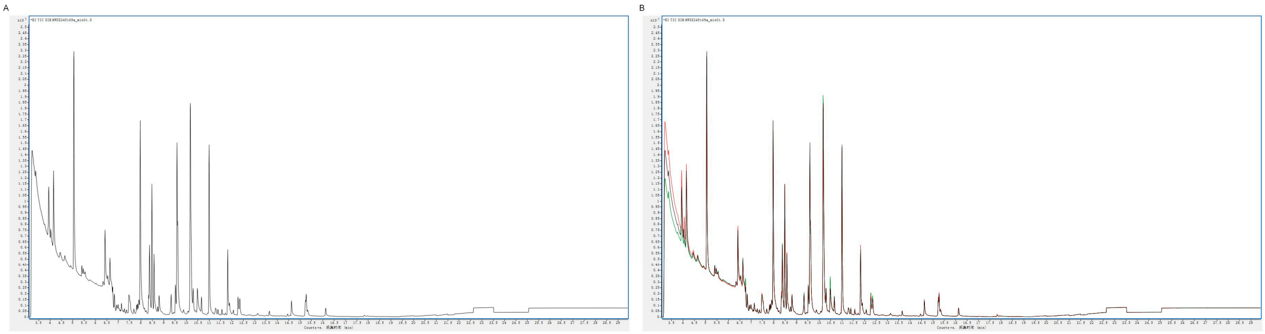

Figure S1: The mass spectrometry analysis of the total ion and the TIC overlap of the QC sample mass spectrum
